# The multi-faceted roles of the PI3K-AKT pathway in melanoma

**DOI:** 10.1186/1479-5876-13-S1-K1

**Published:** 2015-01-15

**Authors:** Michael A Davies

**Affiliations:** 1 Division of Cancer Medicine, The University of Texas MD Anderson Cancer Center, Houston, Texas, USA

## Background

The PI3K-AKT pathway has been shown to complement activation of the MAPK pathway in melanocyte transformation in preclinical models. We have utilized clinical specimens and cell lines to examine the regulation, functional role, and clinical significance of the PI3K-AKT pathway in melanoma.

## Materials and methods

All analyses of clinical specimens were performed under protocols approved by the MD Anderson Institutional Review Board. Identities of cell lines were confirmed by STR-fingerprinting, and cell lines were genotyped by sequenom analysis for hotspot mutations. Reverse phase protein array (RPPA) analysis was performed by the MD Anderson RPPA Core Facility.

## Results

Proteomic analysis of frozen melanoma clinical specimens and cell lines demonstrated that phosphorylation (activation) of AKT (P-AKT) correlated inversely with PTEN protein expression. Confirmatory analysis of PTEN expression by an immunohistochemical (IHC) assay on archival tumor samples identified a linear correlation between PTEN measurement by IHC and RPPA. Significantly increased expression of P-AKT was observed in melanomas with complete loss of PTEN protein expression compared to tumors with any other pattern of PTEN expression. Integrated analysis of BRAF/NRAS mutation status, PTEN expression, and clinical outcomes was performed using lymphadenectomy specimens from patients who underwent surgery for stage IIIB/C melanoma. Complete loss of PTEN expression correlated with shorter overall survival and time to brain metastasis on univariate and multivariate analyses. Testing of human melanoma cell lines demonstrated that cells with baseline or compensatory activation of the PI3K-AKT pathway were generally resistant to apoptosis induction following BRAF or MEK inhibition. Extended molecular profiling found that a subset of melanoma cell lines with de novo and acquired resistance to MEK inhibitors are characterized by increased expression of PGC1alpha and a metabolic phenotype of high oxidative phosphorylation (OxPhos). The resistant high OxPhos cell lines all demonstrated synergistic growth inhibition and apoptosis induction with combined targeting of MEK and mTORC1/2. mTORC1/2 inhibition decreased PGC1alpha expression in vitro and in vivo, and caused cytosolic localization of MITF. Treatment with a MEK inhibitor and an mTORC1/2 inhibitor demonstrated marked synergy in a BRAF-mutant, MEK inhibitor resistant human melanoma cell line in vivo.

## Conclusions

Activation of the PI3K-AKT pathway correlates with clinical outcomes and resistance to MAPK pathway inhibitors in melanoma. These findings support the rationale to determine the clinical benefit of inhibiting the PI3K-AKT pathway in this disease.

